# A Phase II Study Demonstrates No Feasibility of Adjuvant Treatment with Six Cycles of S-1 and Oxaliplatin in Resectable Esophageal Adenocarcinoma, with ERCC1 as Biomarker for Response to SOX

**DOI:** 10.3390/cancers13040839

**Published:** 2021-02-17

**Authors:** Charlotte I. Stroes, Sandor Schokker, Remco J. Molenaar, Ron A. A. Mathôt, Maarten F. Bijlsma, Stephanie O. van der Woude, João P. Belo Pereira, Gerrit K. J. Hooijer, Rob H. A. Verhoeven, Annemieke Cats, Cecile Grootscholten, Johanna W. van Sandick, Geert-Jan Creemers, Grard A. P. Nieuwenhuijzen, Nadia Haj Mohammad, Jelle P. Ruurda, Sybren L. Meijer, Maarten C. C. M. Hulshof, Mark I. van Berge Henegouwen, Hanneke W. M. van Laarhoven

**Affiliations:** 1 Cancer Center Amsterdam, Department of Medical Oncology, Amsterdam UMC, University of Amsterdam, 1105 AZ Amsterdam, The Netherlands; c.i.stroes@amsterdamumc.nl (C.I.S.); s.schokker@amsterdamumc.nl (S.S.); r.j.molenaar@amsterdamumc.nl (R.J.M.); m.f.bijlsma@amsterdamumc.nl (M.F.B.); s.o.vanderwoude@amsterdamumc.nl (S.O.v.d.W.); 2 Center for Experimental and Molecular Medicine (CEMM), Cancer Center Amsterdam, Laboratory for Experimental Oncology and Radiobiology (LEXOR), Amsterdam UMC, University of Amsterdam, 1105 AZ Amsterdam, The Netherlands; 3 Department of Hospital Pharmacy and Clinical Pharmacology, Amsterdam UMC, University of Amsterdam, 1105 AZ Amsterdam, The Netherlands; r.mathot@amsterdamumc.nl; 4 Oncode Institute, 3521 AL Utrecht, The Netherlands; 5 Department of Vascular Medicine, Amsterdam UMC, University of Amsterdam, 1105 AZ Amsterdam, The Netherlands; j.p.belopereira@amsterdamumc.nl; 6 Department of Pathology, Amsterdam UMC, University of Amsterdam, 1105 AZ Amsterdam, The Netherlands; g.k.hooijer@amsterdamumc.nl (G.K.J.H.); s.l.meijer@amsterdamumc.nl (S.L.M.); 7 Department of Research and Development, Netherlands Comprehensive Cancer Organization (IKNL), 3511 DT Utrecht, The Netherlands; r.verhoeven@iknl.nl; 8 The Netherlands Cancer Institute—Antoni van Leeuwenhoek Hospital, Department of Gastrointestinal Oncology, 1066 CX Amsterdam, The Netherlands; a.cats@nki.nl (A.C.); c.grootscholten@nki.nl (C.G.); 9 Department of Surgery, The Netherlands Cancer Institute—Antoni van Leeuwenhoek Hospital, 1066 CX Amsterdam, The Netherlands; j.v.sandick@nki.nl; 10 Department of Medical Oncology, Catharina Hospital, 5623 EJ Eindhoven, The Netherlands; geert-jan.creemers@catharinaziekenhuis.nl; 11 Department of Surgery, Catharina Hospital, 5623 EJ Eindhoven, The Netherlands; grard.nieuwenhuijzen@catharinaziekenhuis.nl; 12 University Medical Center Utrecht, Department of Clinical Oncology, Utrecht University, 3584 CX Utrecht, The Netherlands; n.hajmohammad@umcutrecht.nl; 13 University Medical Center Utrecht, Department of Surgery, 3584 CX Utrecht, The Netherlands; j.p.ruurda@umcutrecht.nl; 14 Department of Radiation Oncology, Amsterdam UMC, University of Amsterdam, 1105 AZ Amsterdam, The Netherlands; m.c.hulshof@amsterdamumc.nl; 15 Department of Surgery, Amsterdam UMC, University of Amsterdam, 1105 AZ Amsterdam, The Netherlands; m.i.vanbergehenegouwen@amsterdamumc.nl

**Keywords:** esophageal adenocarcinoma, adjuvant chemotherapy, S-1, oxaliplatin, predictive biomarkers, S-1 pharmacokinetics, proteomics

## Abstract

**Simple Summary:**

Neoadjuvant chemoradiotherapy followed by surgery is currently standard of care in esophageal adenocarcinoma. However, prognosis remains dismal. The aim of our study was to assess the feasibility of administering six cycles of adjuvant S-1 and oxaliplatin following neoadjuvant chemoradiotherapy and esophagectomy. Although six cycles of adjuvant S-1 and oxaliplatin were not feasible in pretreated patients, mainly due to toxicity, efficacy results were promising compared to a propensity-score matched cohort. Exploratory biomarker analyses demonstrated potential benefit for patients with Excision repair cross-complementation group 1 (ERCC1) negative tumor expression. A proteomics biomarker model provided valuable information for prediction of survival and pharmacokinetics of 5-FU showed a correlation with treatment-related toxicity. Although it remains unclear if additional chemotherapy should be provided in the adjuvant setting, subgroups such as patients with ERCC1 negativity, could potentially benefit from this treatment option based on our exploratory biomarker research.

**Abstract:**

We assessed the feasibility of adjuvant S-1 and oxaliplatin following neoadjuvant chemoradiotherapy (nCRT) and esophagectomy. Patients treated with nCRT (paclitaxel, carboplatin) and esophagectomy received six 21-day cycles with oxaliplatin (130 mg/m^2^) on day 1 and S-1 (25 mg/m^2^ twice daily) on days 1–14. The primary endpoint was feasibility, defined as ≥50% completing treatment. We performed exploratory propensity-score matching to compare survival, ERCC1 and Thymidylate Synthase (TS) immunohistochemistry analyses, proteomics biomarker discovery and 5-FU pharmacokinetic analyses. Forty patients were enrolled and 48% completed all adjuvant cycles. Median dose intensity was 98% for S-1 and 62% for oxaliplatin. The main reason for early discontinuation was toxicity (67%). The median recurrence-free and overall survival were 28.3 months and 40.8 months, respectively (median follow-up 29.1 months). Survival was not significantly prolonged compared to a matched cohort (*p* = 0.09). Patients with ERCC1 negative tumor expression had significantly better survival compared to ERCC1 positivity (*p* = 0.01). Our protein signature model was predictive of survival [*p* = 0.04; Area under the curve (AUC) 0.80]. Moreover, 5-FU pharmacokinetics significantly correlated with treatment-related toxicity. To conclude, six cycles adjuvant S-1 and oxaliplatin were not feasible in pretreated esophageal adenocarcinoma. Although the question remains whether additional treatment with chemotherapy should be provided in the adjuvant setting, subgroups such as patients with ERCC1 negativity could potentially benefit from adjuvant SOX based on our exploratory biomarker research.

## 1. Introduction

Esophageal cancer is the 7th most common type of cancer worldwide, with 572,000 newly diagnosed patients in 2018 [1]. It ranks sixth in cancer mortality, accounting for over 509,000 deaths annually. In the Western World, neoadjuvant chemoradiotherapy (nCRT) or chemotherapy followed by esophagectomy is the current standard of care in resectable esophageal adenocarcinoma (EAC) [2,3]. Despite the benefit achieved with multimodality treatment, recurrence rates as distant metastases remain high following curative treatment. Thus, further optimization of systemic treatment is urgently needed [1,2].

Although multiple studies investigated adjuvant regimens to decrease postoperative recurrence in gastrointestinal cancer, benefit in EAC could not be confirmed as randomized trials are lacking [4,5]. In patients with resected gastric cancer, adjuvant capecitabine and oxaliplatin (CAPOX) significantly improved disease-free survival, compared to surgery alone [6]. Significant survival benefit was also reported in Asian gastric cancer patients treated with adjuvant S-1 (an oral fluoropyrimidine) following surgery, compared to surgery alone [7]. The combination of adjuvant S-1 and oxaliplatin (SOX) demonstrated non-inferiority compared to adjuvant CAPOX in gastrointestinal cancer, with significantly lower incidences of hematological toxicity and hand-foot syndrome [8,9]. However, it should be noted that none of the latter studies included patients who received preoperative treatment. 

Given that S-1 demonstrated a favorable toxicity profile and SOX has shown tolerability and efficacy in gastrointestinal cancer, we primarily assessed the feasibility of administering six cycles of adjuvant SOX in patients with EAC who were pretreated with nCRT and esophagectomy [7,8,10].

As EAC is a heterogenic disease, the current focus is directed towards a personalized treatment approach. Therefore, we aimed to exploratory identify subgroups with most benefit of SOX treatment. Currently, definite biomarkers are lacking for EAC [11]. Therefore, we first investigated the potential role of Excision-repair cross-complementation group 1 (ERCC1) and Thymidylate Synthase (TS) as immunohistochemical biomarkers for response to SOX, as both markers have previously been identified to play a role in demonstrating response to platinum and 5-FU treatment, respectively [12,13]. Secondly, we utilized targeted protein biomarker discovery to gain insight into the underlying resistance mechanisms, as well as to identify subgroups with most benefit of adjuvant SOX on protein level. 

Since ethnical differences in S-1 metabolism and efficacy have been identified previously, we exploratory assessed the 5-FU pharmacokinetics to gain insight in the concentrations achieved. Moreover, as major surgery could potentially affect 5-FU pharmacokinetics, we aimed to assess the effect of esophagectomy on the S-1 metabolite 5-FU [14].

## 2. Results

### 2.1. Baseline Characteristics

Forty patients were enrolled from three centers in the Netherlands between February 2015 and May 2018 to receive six cycles of adjuvant S-1 and oxaliplatin. The median age was 61 years (interquartile range (IQR) 54–64) and the majority of patients were male (*n* = 37, 93%). Most patients had tumors located in the distal esophagus (73%) and were staged ypT3 (58%; Table 1).

### 2.2. Exposure to Treatment

Nineteen of 40 patients (48%) completed all six cycles of SOX. Twenty-one patients did not receive full preplanned treatment due to toxicity (*n* = 14, of which 57% was peripheral sensory neuropathy), patient’s request (*n* = 5) or recurrence of disease (*n* = 2). Thirty patients (75%) completed six cycles with S-1. The median dose intensity of the entire cohort was 98% (IQR 73–100%) for S-1 and 62% (IQR 46–83%) for oxaliplatin. For oxaliplatin, doses were reduced in 26 patients (65%), delayed in 11 patients (28%) and interrupted in one patient (3%). For S-1, doses were reduced in three patients (8%), delayed in 13 patients (33%) and interrupted in seven patients (18%; Figure S1). The majority of patients (75%) completed four of the six preplanned cycles.

### 2.3. Safety

The most common grade 1/2 toxicities included fatigue (88%), neurosensory toxicity (75%), nausea (58%) and diarrhea (58%; Table 2). Four patients (10%) experienced hand-foot syndrome. Nausea (18%) and peripheral sensory neuropathy (13%) were the most common grade ≥3 toxicities. There were no treatment-related deaths. Four serious adverse events (SAEs) were recorded in four patients (10%); fever, nausea, cholangitis and pneumonia. All patients recovered from these SAEs, although it resulted in early trial discontinuation of two patients. 

### 2.4. Survival

**Figure 1:**
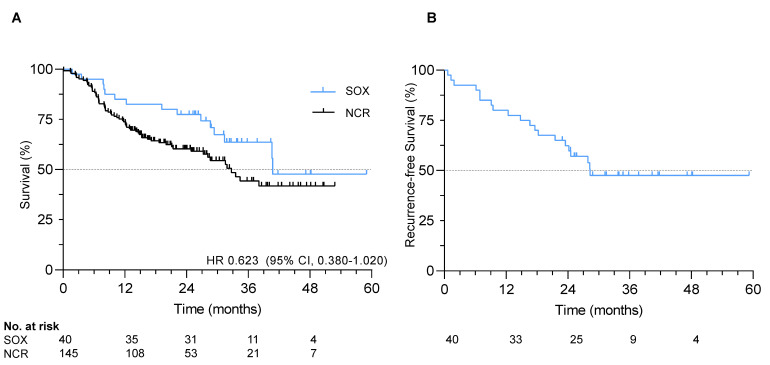
Survival in the S-1 and oxaliplatin (SOX) study. (**A**) Overall survival in the SOX cohort and exploratory comparative survival with a propensity-score matched cohort; (**B**) Recurrence-free survival in the SOX study.

At data cut-off in January 2020, median follow-up was 29.1 months (IQR 23.3–37.3). The median recurrence-free survival (RFS) and overall survival (OS) were 28.3 and 40.8 months, respectively. One-, two- and three-year RFS rates were 80%, 62% and 48%, respectively. One-, two- and three-year OS rates were 85%, 78% and 64%, respectively (Figure 1). 

All 40 patients were matched to 145 patients from the Netherlands Cancer Registry (Table S1). No statistically significant difference in hazard of death was observed in patients receiving adjuvant SOX compared to the matched cohort receiving nCRT only (HR 0.623, 95% CI 0.380–1.020, *p* = 0.09), although numerically survival was superior with SOX (median OS 40.8 months vs. 32.7 months; Figure 1). Median follow-up in the matched cohort was significantly shorter with 17.8 months (*p* < 0.001).

In subgroup analysis, patients with well or moderately differentiated tumors and four or more resected positive lymph nodes had significantly longer OS with adjuvant SOX compared to nCRT and esophagectomy only (*p* = 0.01; *p* = 0.05 respectively; Figure S2).

### 2.5. ERCC1 as Potential Predictive Biomarker

**Figure 2:**
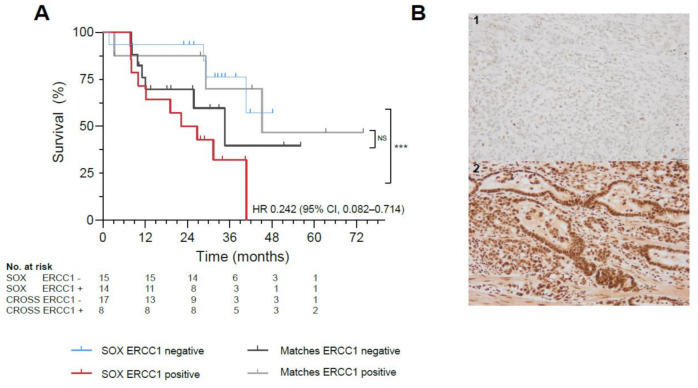

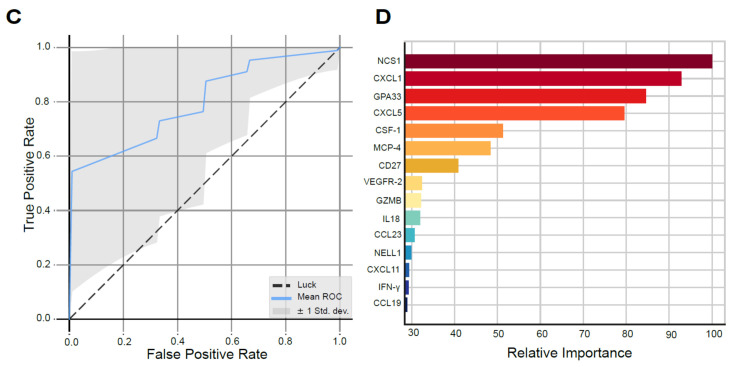
Biomarker Analyses in the SOX study. (**A**) Significant survival benefit in patients with ERCC1 negative resection specimens from the SOX cohort (blue) vs. ERCC1 positive resection specimens (red, *** indicates *p*-value of 0.008) compared to a matched cohort receiving standard neoadjuvant chemoradiotherapy with ERCC1 negative resection specimens (black) vs. ERCC1 positive resection specimens (gray, NS indicates not significant). (**B**)Representative illustrations of ERCC1 negative (1) and ERCC1 positive (2) tumors at 20x magnification. (**C**) AUC of the proteomics model, with a mean ROC AUC of 0.80 (standard deviation 0.22). (**D**) Fifteen most important proteins in the proteomics model predictive for survival in the SOX cohort, ranked from highest relative importance to lowest relative importance.

Twenty-nine biopsies and resections were evaluable for analyses, as not all tissues could be retrieved and four patients had a complete response following nCRT (Supplementary Materials S1.4). Patients with ERCC1 negative resection specimens (*n* = 15) demonstrated significantly better OS, compared with ERCC1 positive resection specimens (*n* = 14) (HR 0.242, 95% CI, 0.082–0.714, *p* = 0.008) (Figure 2A,B, Figure S3). No correlation between survival benefit and ERCC1 negative tumor expression was observed in a matched cohort treated with standard nCRT (*p* = 0.558; Figure 2A, Table S2), potentially indicating a predictive role for ERCC1 as biomarker of response to SOX. In primary tumor biopsies, ERCC1 negativity did not correlate to survival (*p* = 0.656).

Patients with TS negative primary tumor biopsies or negative resection specimens did not demonstrate a significantly better OS, compared to TS positivity (*p* = 0.100; *p* = 0.930 respectively; Figures S4 and S5).

### 2.6. Protein Signature Model Predictive for Survival

Thirty-nine patients were included, as plasma of one patient was missing. Using machine learning, we identified a signature model of 15 plasma proteins significantly predictive of survival with SOX (*p* = 0.039). The protein model resulted in a ROC with an AUC of 0.80 (SD 0.22; Odds Ratio 8.58, Figure 2C). The six proteins with the highest relative importance in the prediction of survival in the protein model were neuronal calcium sensor-1 (*NCS1*), chemokine ligand 1 (*CXCL1*), glycoprotein A33 (*GPA33*), chemokine ligand 5 (*CXCL5*) and colony stimulating factor 1 (*CSF-1*), respectively (Figure 2D). The full proteomics expression data can be found supplementary (Supplementary Proteomics Data file; Table S3, Figure S6).

### 2.7. Pharmacokinetic Analysis of 5-FU

**Figure 3:**
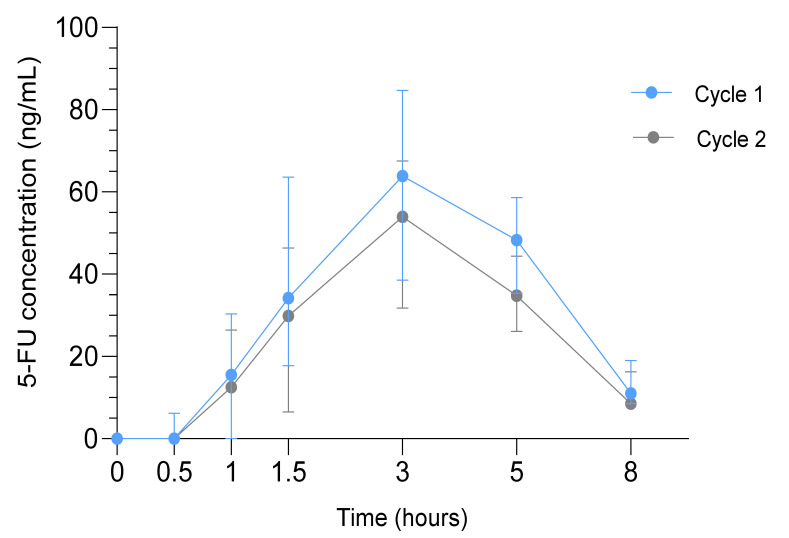
Pharmacokinetic Analysis in the SOX study. Median 5-FU concentrations in ng/mL (interquartile range) in cycle 1 (blue) and cycle 2 (gray) at t = 0 to t = 8 h after drug administration.

The median AUC_0-∞_ (range) of 5-FU was significantly higher in cycle 1 (389.6 ng/mL*h (228.1–663.6), *n* = 39) compared to cycle 2 (308.9 ng/mL*h (114.8–457.9), *n* = 35; *p* < 0.001). The median C_max_ (range) in cycle 1 was 71.6 ng/mL (19.0–166.0) and 62.0 ng/mL (25.2–107.0) in cycle 2 (*p* = 0.002). The median T_max_ and T_1/2_ were 3 h and 1.7 h in both cycles, respectively (Figure 3). 

Higher C_max_ and AUC_0-∞_ of 5-FU correlated with higher toxicity grades of diarrhea (*p* = 0.05, *p* = 0.001 respectively), nausea (*p* = 0.03, *p* = 0.04 respectively) and vomiting (*p* = 0.02, *p* = 0.04 respectively). No correlations were observed between pharmacokinetics and recurrence or survival.

## 3. Discussion

This is, to our knowledge, the first prospective study investigating the feasibility of adjuvant chemotherapy with SOX in the treatment of EAC following nCRT and surgery. Unfortunately, six cycles of adjuvant SOX were not feasible (completion rate 48%).

The majority of patients could not complete six cycles of adjuvant SOX. Multiple studies investigating adjuvant therapy with CAPOX or SOX regimens in resected gastroesophageal cancer patients demonstrated higher completion rates (66–74%) [6,7,16]. However, patients included in the aforementioned studies did not receive preoperative chemoradiotherapy. The lower likelihood of completing adjuvant treatment following pretreatment prior to surgery has been reported [17]. This is supported by the low completion rates of postoperative treatment (42–50%) achieved in studies with gastroesophageal cancer patients assigned to both pre- and postoperative treatment, which are in line with our completion rate [18,19,20]. In colorectal cancer, non-inferior survival and higher completion rates with less toxicity were reported with three months adjuvant CAPOX, compared to six months [21]. Extrapolating these data to our patient population, it could be hypothesized that four cycles of adjuvant SOX would provide similar results in terms of efficacy compared to six cycles, while based on our results this would in fact be feasible.

We observed a toxicity profile similar to other studies administering SOX [16,22]. Nevertheless, toxicity was significant and resulted in dose reductions in 65% of patients. Although the incidence of oxaliplatin-related sensory neuropathy was in the same line as in other studies, neuropathy was the contributing factor for 38% of all early trial discontinuations [16,22]. Hematological toxicity in our study was reported less frequently compared to Asian studies administering SOX, while the incidence of diarrhea was slightly higher [10,16,23]. These findings reflect the ethnical differences in the toxicity profile of S-1 previously reported, with predominantly hematological toxicity in Asians and gastrointestinal toxicity in the Western world [24,25,26]. Moreover, it underlines the previously identified difference in S-1 metabolism and tolerability between both populations, which is also reflected in the lower maximum tolerated dose in the Western world [27].

Although less than half of the patients received all preplanned cycles, exploratory efficacy data were promising. This can potentially be contributed to the high S-1 dose intensity achieved. Survival of patients who received additional SOX was numerically longer compared to the matched cohort receiving nCRT and surgery only, albeit not statistically significant. As the median follow-up was significantly shorter in the matched cohort, survival benefit might become more evident with longer follow-up. Although both cohorts were matched, this was a non-randomized analysis and selection bias could have played a role by comparing trial patients to real world data.

Our subgroup analysis indicates potential benefit of adjuvant SOX in patients with four or more resected positive lymph nodes and well or moderately differentiated tumors. This supports the assumption that benefit of adjuvant treatment with chemotherapy is most evident in patients with residual nodal disease following surgery. Benefit of adjuvant treatment in patients with four or more positive nodes has been demonstrated in larger studies [4,28,29]. Although the predictive value of low differentiation grade could contradict the notion of most benefit of adjuvant chemotherapy in patients with higher tumor aggressiveness, it has previously been reported as predictive factor for additional treatment in esophageal cancer [30]. We identified no significant benefit of SOX over standard treatment in subgroups with high vs. low Mandard or high vs. low T-stage. Unfortunately, adequately powered studies on predictive factors for adjuvant treatment in EAC are lacking. Therefore, our findings need further confirmation.

To further explore potential predictive biomarkers, we analyzed the expression of ERCC1 and TS with immunohistochemistry. ERCC1 plays a critical role in the repair of platinum-induced DNA damage and has been reported as a negative predictive marker for response to platinum treatment in various cancer types [12,31]. Similarly, in our cohort patients with ERCC1 negative tumors demonstrated significantly better survival compared to patients with ERCC1 positivity. In the validation cohort including patients treated with nCRT only, no correlation between ERCC1 negativity and survival benefit was observed. Therefore, ERCC1 could potentially be a predictive biomarker for SOX treatment. 

TS was reported as negative predictive biomarker of response to 5-FU and S-1 treatment but we found no correlation between TS expression and survival [13]. Although immunohistochemistry analyses were performed on a small cohort, these findings suggest that the absence of ERCC1 tumor expression holds a prognostic and potentially predictive value as biomarker in response to adjuvant SOX.

Nevertheless, esophageal cancer remains a heterogenic disease and previous research demonstrated limited value of single biomarkers for all patients. Therefore, the current focus is directed towards multi-biomarker signatures [32]. Using proteomics, we found a biomarker model identifying patients with better survival at high diagnostic accuracy. In this model, 15 proteins with previously reported importance in cancer pathogenesis demonstrated the highest relevance. For instance, *NCS1* and *CSF-1* are associated with promotion of tumor aggressiveness and poor outcome in various cancer types [33,34]. Furthermore, *GPA33* is overexpressed in >95% of colon cancers and >60% of gastric cancers and is identified as a Barrett metaplasia marker [35,36]. Both *GPA33* and *CSF-1* show promising potential for targeted anti-cancer treatment [37,38]. This model provides a promising biomarker in EAC, which currently lacks definite biomarkers. Although this analysis was limited by the sample size, cross-validation and data sub-sampling were performed to prevent overfitting. However, it cannot be discriminated if this protein signature holds prognostic or also predictive value for SOX treatment. Investigating this model in an EAC cohort receiving nCRT could provide further insight.

In pharmacokinetic analyses of 5-FU, we found lower concentrations compared to 5-FU pharmacokinetic studies in gastric cancer [39,40]. This is not attributable to oxaliplatin co-administration, as this has been shown not to affect 5-FU concentrations [41]. Moreover, dose-dependency is unlikely to play a role, as concentrations were also lower when equal doses were administered [24,25]. However, the majority of previous studies were performed in Asia and ethnical differences in 5-FU pharmacokinetics between the population in Asia and the Western world were identified previously [24]. For example, ethnical differences in the CYP2A6 enzyme have been demonstrated, leading to differences in tegafur metabolism [42]. Moreover, esophagectomy could have impacted 5-FU pharmacokinetics, as surgery could change intestinal absorption [43,44]. Indeed, pharmacokinetic changes of S-1 have been observed following gastrectomy, although differences were small [14].

Additionally, we observed a significant decrease of 5-FU in cycle 2 compared to cycle 1, without dose modifications. The clinical relevance and reason behind this difference currently remain unknown. Additional benefit of increasing the S-1 dose to circumvent this decrease is not advisable in combination with oxaliplatin, as we found a correlation between 5-FU concentrations and potential S-1 related toxicities [9,24,25].

As efficacy results of our study were promising, it could be hypothesized that adjuvant treatment with chemotherapy may be beneficial. However, given the limited tolerability, further research on adjuvant treatment should focus on subgroups of patients with most benefit of adjuvant treatment. Moreover, as survival benefit has been demonstrated with induction chemotherapy prior to nCRT, the question remains if additional treatment with chemotherapy should be provided in the adjuvant setting for esophageal cancer [45].

## 4. Materials and Methods 

### 4.1. Patient Eligibility Criteria

Eligible patients completed nCRT with paclitaxel 50 mg/m^2^, carboplatin AUC = 2 and 23 × 1.8 Gray radiotherapy (CROSS regimen) and had macroscopic radically resected EAC [2]. Key inclusion criteria were an Eastern Cooperative Oncology Group score of 0/1, no metastases and adequate bone marrow, renal and hepatic functions (Supplementary Materials S1.1). 

This study was approved by the institutional review board of the Amsterdam University Medical Center (location Academic Medical Center) and conducted in accordance with Good Clinical Practice and the Declaration of Helsinki. All patients provided written informed consent.

### 4.2. Study Design

This was a prospective, multicenter, phase II study (trial registration number NCT02347904) investigating the feasibility of six cycles of adjuvant SOX in patients following nCRT and esophagectomy. Within 16 weeks following esophagectomy, patients started with six 21-day cycles consisting of oxaliplatin 130 mg/m^2^ intravenously on day 1 and S-1 25 mg/m^2^ twice daily orally (total 50 mg/m^2^) on days 1–14. Treatment was discontinued in case of disease recurrence or unacceptable toxicity.

### 4.3. Dose Modifications

Toxicity was graded using the National Cancer Institute Common Terminology Criteria for Adverse Events version 4.3. In case of toxicity, doses were reduced per judgement of the treating physician, using the de-escalation steps specified in the protocol (Supplementary Materials S1.2). For S-1 and oxaliplatin, dose re-escalation was not allowed. Dose interruptions for recovery were allowed up until 14 days.

### 4.4. Study End Points and Statistics

The primary objective was to assess feasibility of treatment with adjuvant SOX in patients with EAC following nCRT and esophagectomy. Feasibility was defined as ≥50% of patients completing six preplanned cycles, based on prior completion rates of S-1 in gastric cancer and CAPOX in colon cancer [7,46]. An exact one-sided binomial test was used for statistics. Based on a single-stage Fleming Design, inclusion of 40 patients would achieve an alpha of 0.05 and power of 80%. 

Secondary endpoints were dose intensity of S-1 and oxaliplatin (total dose received divided by total dose planned ×100%), completion of S-1, dose modifications (reductions, interruptions, delays >3 days), safety and recurrence-free (RFS) and overall survival (OS). Safety and survival analyses included all patients who received at least one dose of SOX. Exploratory endpoints were subgroup analyses, ERCC1 and TS expression and proteomics biomarker discovery analysis in relation to efficacy and 5-FU pharmacokinetics in relation to safety and efficacy. 

All tests were two-sided, with *p*-values < 0.05 considered statistically significant. SPSS Version 26.0 (Armonk, NY, USA: IBM Corp.) was used for statistics. 

#### 4.4.1. Survival

RFS and OS were estimated using Kaplan-Meier analyses, measured from the date of first SOX administration to documented recurrence or death of any cause, respectively. In an exploratory analysis, we performed propensity-score matching with data from the Netherlands Cancer Registry to compare OS, matching SOX patients to patients receiving nCRT and esophagectomy using a logistic regression model on prespecified patient and tumor characteristics (Supplementary Materials S1.3). In the matched cohort, follow-up was measured from 16 weeks following esophagectomy. Patients were matched 1:4 with a maximum propensity-score difference of 0.20, using greedy-neighbor matching. Survival was analyzed using a Cox proportional hazards model.

Exploratory multivariate subgroup analyses were performed with a Cox proportional hazards model using preselected prognostic factors in EAC[47]. The propensity-score matched cohort served as a control arm. A *p*-value for interaction <0.05 was considered statistically significant.

#### 4.4.2. Immunohistochemistry

Paraffin-embedded tumor samples from treatment-naïve primary tumor biopsies and resection specimens were stained for TS and ERCC1 using monoclonal antibodies against TS (TS 106) and ERCC1 (8F1). Archival resection specimens from the Pathology department of the Amsterdam UMC, location AMC, of a cohort treated with nCRT only were stained for ERCC1, matched to the SOX cohort on age, sex, ypT, ypN and Mandard score. Slides were scored by a certified pathologist using the H-score, calculated by multiplying the staining intensity with the concomitant percentage of positive tumor cells. ERCC1 and TS staining intensities were scored from 0–3 and 0–4, respectively, with higher scores indicating higher intensities [48,49]. The median was used as cut-off to define positivity (Supplementary Materials S1.4).

#### 4.4.3. Proteomics

Plasma samples from day 1 of cycle 1 or cycle 2 were analyzed by Olink Proteomics AB (Uppsala, Sweden). Briefly, concentrations of 184 proteins were assessed using the 92-plex proximity-extension assay using the immune-oncology panel and oncology panel III. In the assay, oligonucleotide-labeled antibody pairs can bind to target proteins. Upon binding of a pair, a polymerase chain reaction (PCR) target sequence is formed, allowing for subsequent detection by high-throughput real-time PCR (Supplementary Materials S1.5) [45]. For the machine learning analysis, the packages Numpy, Scikit-learn and Scipy were used in Python version 3.7. We applied XGBoost, a gradient boosting framework, with multiple levels of gradient boosting classifiers to identify a model predictive of survival [46,47]. Eighty percent of data was used as training set and a 10-fold stratified cross-validation was applied. We conducted a 50-fold rigorous stability selection procedure, resulting in a receiver operating characteristics curve (ROC) AUC [48]. To ensure model reliability, a permutation test was performed in which the outcome (OS) was reshuffled for 1000 times, whilst the protein patterns remained stable.

#### 4.4.4. Pharmacokinetics

Plasma samples were obtained on day 1 of cycle 1 and 2 before treatment administration and at 0.5, 1, 1.5, 3, 5 and 8 h thereafter. The active metabolite of S-1, 5-fluorouracil (5-FU), was isolated from plasma and concentrations were analyzed using liquid chromatography-mass spectrometry (Supplementary Materials S1.6). Noncompartmental pharmacokinetic analyses were performed using PKSolver [50]. The maximum concentration (C_max_) and time to reach C_max_ (T_max_) were determined and the AUC from t = 0 to t = ∞ was calculated with a linear trapezoidal method. Nonparametric paired tests were applied for statistics.

## 5. Conclusions

In conclusion, administration of six cycles of adjuvant SOX was not feasible in EAC patients pretreated with nCRT and surgery, mainly due to toxicity. Despite the low completion rates, efficacy results were promising but the survival benefit achieved was not significant compared to a propensity-score matched cohort. ERCC1 may serve as biomarker to predict survival and potentially response to SOX. Further research should focus on providing four cycles of adjuvant treatment to specific subgroups of patients with most benefit based on biomarker research, such as patients with ERCC1 negativity.

## Figures and Tables

**Table 1:** Baseline Characteristics of all included patients (*N* = 40). IQR denotes interquartile range.

Characteristic	*N* = 40	
	No. of Patients	%
Sex		
Male	37	93
Female	3	8
Age, years		
Median	61	
IQR	54–64	
Tumor location		
Lower thoracic	29	73
Esophagogastric junction	11	28
Tumor differentiation grade		
1	2	5
2	21	53
3	14	35
4	2	5
*x*	1	3
Type of resection		
Transthoracic with intrathoracic anastomosis	33	83
Transthoracic with cervical anastomosis	4	10
Transhiatal	3	8
ypT classification		
0	4	10
1	7	18
2	6	15
3	23	58
ypN classification		
0	19	48
1	14	35
2	4	10
3	3	8
Radicality of resection		
R0	39	98
R1	1	3
Mandard score [15]		
1	4	10
2	13	33
3	17	43
4	6	15
5	0	0

**Table 2:** Reported Adverse Events of grade 1–2 toxicities occurring in ≥10% of patients and all grade ≥3 toxicities, graded according to the Common Terminology Criteria for Adverse Events v. 4.3. Skin toxicity was acneiform rash (*N* = 3, 8%), dermatitis (*N* = 1, 3%) and aggravation of psoriasis (*N* = 1, 3%). ALP denotes alkaline phosphatase; AST denotes aspartate aminotransferase.

Adverse Event	Grade 1–2		Grade ≥ 3	
Caption	No. of Patients	%	No. of Patients	%
Hematological Toxicity				
Thrombocytopenia	6	15	0	0
Gamma-GT increase	5	13	1	3
ALP increase	5	13	0	0
AST increase	4	10	0	0
Non-hematological Toxicity				
Fatigue	35	88	1	3
Peripheral sensory neuropathy	30	75	5	13
Nausea	23	58	7	18
Diarrhea	23	58	1	3
Anorexia	16	40	3	8
Vomiting	14	35	3	8
Constipation	12	30	1	3
Injection-site reaction	13	33	0	0
Malaise	12	30	0	0
Dyspnea	10	25	0	0
Dysphagia	8	20	0	0
Abdominal pain	6	15	1	3
Cough	7	18	0	0
Reflux disease	7	18	0	0
Pain	6	15	0	0
Laryngospasm	5	13	0	0
Muscle cramps	5	13	0	0
Peripheral motor neuropathy	5	13	0	0
Skin toxicity	5	13	0	0
Dizziness	4	10	0	0
Dysesthesia	3	8	1	3
Hand-foot syndrome	4	10	0	0
Headache	4	10	0	0
Insomnia	4	10	0	0
Gastrointestinal hemorrhage	4	10	0	0
Mucositis	4	10	0	0
Fever	2	5	1	3
Pneumonia	1	3	1	3
Cholangitis	0	0	1	3
Thrombo-embolic event	0	0	1	3

## Data Availability

The data presented in this study are partially available in the Supplementary Materials. All data are available on reasonable request from the corresponding author.

## Supplementary Materials

The following are available online at https://www.mdpi.com/2072-6694/13/4/839/s1, Figure S1: Treatment Exposure of S-1 and oxaliplatin, Figure S2: Multivariate Subgroup Analysis in the SOX study, Figure S3: ERCC1 immunohistochemistry expression at 20x magnification, Figure S4: TS immunohistochemistry expression at 20x magnification, Figure S5: Survival of patients with TS negative resection specimens vs. TS positive resection specimens, Figure S6: Heatmap of differentially expressed proteins between dead vs. alive patients, Table S1: Propensity-score Matching Details of the SOX cohort and the Netherlands Cancer Registry cohort, Table S2: Details of the SOX cohort and the matched cohort for ERCC1 immunohistochemistry, Table S3: Description of the proteins included in the machine learning model. Supplementary Excel file with Proteomics data.
