# Supplementary material for: A Phase II Study Demonstrates No Feasibility of Adjuvant Treatment with Six Cycles of S-1 and Oxaliplatin in Resectable Esophageal Adenocarcinoma, with ERCC1 as Biomarker for Response to SOX
Source: Cancers (Basel). 2021 Feb 17;13(4):839. doi: 10.3390/cancers13040839 (PMC7922275; doi:10.3390/cancers13040839)
Supplement: Supplementary file 1 [file cancers-13-00839-s001.zip › cancers-1022378 Supplementary Appendix SOX study.pdf]

Supplementary Appendix

Appendix 1: Supplementary Methods p. 2

Appendix 2: Supplementary Figures p. 6

Appendix 3: Supplementary Tables p. 11

Appendix 1: Supplementary Methods

1.1 Detailed Inclusion Criteria

Patients were allowed to have a maximum of one missed dose of neoadjuvant systemic therapy due to non-hematological toxicity. Patients required to be ≥18 years, and women with childbearing potential required a negative pregnancy test. Adequate bone marrow function was defined as Hb ≥ 6 mmol/L, absolute neutrophil count ≥ 1.0 x 10^9/L, platelets ≥ 100 x 10^9/L; adequate renal functions were defined as serum creatinine ≤ 1.5x upper limit of normal and creatinine clearance ≥30 mL/min, and adequate hepatic functions were defined as serum bilirubin ≤ 2x and serum transaminases ≤ 3x upper limit of normal. Tumor regression of patients was scored according to the Mandard score.[1] At initiation of the study, patient were enrolled if they were fit enough to start adjuvant treatment within 12 weeks after esophagectomy. After protocol amendment, this inclusion criteria was extended to 16 weeks following surgery.

Patients were excluded on base on any history or clinical signs of metastases, past or current malignancy other than entry diagnosis, significant cardiovascular disease within one year before start of study as determined by the investigator, chronic active infection or concurrent severe or uncontrolled disease preventing the safe administration of study drugs, any impairment of gastrointestinal function or disease that may significantly impair the absorption of oral drugs, known DPD-deficiency or treatment within 4 weeks with DPD-inhibitors, concomitant treatment within 4 weeks before the start of the study of any other experimental drug under investigation or concurrent treatment with any other anti-cancer therapy, or continuous use of systemic immunosuppressive agents (except the use of corticosteroids as anti-emetic prophylaxis of treatment).

1.2 Dose Modifications

If body weight changed >10%, doses were recalculated. The dose of S-1 was reduced to 80% at the second occurrence of a grade 2 toxicity, or the first occurrence of a grade 3 toxicity.

A dose reduction to 60% was applied in case of a third occurrence of a grade 2 toxicity, a second occurrence of a grade 3 toxicity, or the first occurrence of a grade 4 toxicity. Treatment with S-1 was discontinued if a grade 2 toxicity occurred for the fourth, a grade 3 for the third, or a grade 4 for the second time, respectively. Interrupted doses of S-1 were not administered afterwards.

Dose reduction of oxaliplatin to 75% applied for grade 2 toxicity or a first occurrence of grade 3 toxicity. In case of a third occurrence of grade 2 toxicity, a second occurrence of grade 3 toxicity, or every occurrence of grade 4 toxicity resulted in a reduces dose of 50%. Neurotoxicity was an exception, with a 25% reduction for grade 1 toxicity, a 50% reduction for grade 2 toxicity, and discontinuation of treatment for grade 3. For hematological toxicities, thrombocytopenia grade 4, neutropenia grade 4, or febrile neutropenia led to a dose reduction to 75% of oxaliplatin and 80% of S-1. Upon the first recurrence, doses of oxaliplatin and S-1 were reduced to 50% and 70%, respectively, and study treatment were discontinued upon recurrences thereafter.

If patients requested dose modifications or treatment discontinuation upon toxicity, which should according to the protocol not have resulted into modifications or discontinuation, this was classified as 'patients' wish' .

1.3 Propensity-score matching

Patients were matched on the following criteria: age, sex, tumor location, tumor length, differentiation grade, type of resection, number of comorbidities, postoperative complications, pathological T stage, pathological N stage, and hospital resection volume. In the greedy nearest neighbor matching technique each SOX patient is sequentially matched with up to four nearest control patients form the Netherlands Cancer Registry, if within the preset caliper range of 0.2. If a pair is matched, they are removed from the set, and the next pair is chosen. As data on recurrence events was not yet present in the Netherlands Cancer Registry, a propensity-score matched analysis could only be performed for OS.

1.4 Immunohistochemistry

Formalin-fixed, paraffin-embedded tumors were sectioned at 4 μm and dried overnight at 37°C. Sections were deparaffinized in Xylene and hydrated gradually through decreasing concentrations of ethanol. Sections were incubated in 0.3% hydrogen peroxidase in methanol for 20 minutes to remove endogenous peroxidase activity.

For the TS (TS 106) staining, antigen retrieval was performed with 10 mM Tris-1 mM EDTA pH 9.0 at 120°C om a pressure cooker, and subsequently incubated overnight at room temperature with primary mouse monoclonal TS antibody (TS 106, Santa Cruz Biotechnology), diluted at 1:50. After washing in PBS-T, sections were incubated with secondary Brightvision poly-anti-Rabbit IgG antibody (immunologic) for 30 minutes at room temperature. Bound antibody was detected using Bright DAB+ detection kit (Immunologic) and slides were counterstained with 1:5 Hematoxylin (Klinipath).

ERCC1 staining was performed using a Ventana Benchmark Ultra autostainer (Ventana Medical Systems, Tucson, AZ). Antigen retrieval was performed with CC1. ERCC1 was detected with an ERCC1 antibody clone 8F1, Thermo Scientific) and the sections were incubated in a 1:100 dilution for 32 minutes at 37°C. Bound antibody was detected using the Biotin free Optiview DAB Detection Kit (Roche Diagnostics) and slides were counterstained with Hematoxylin.

The H-score for ERCC1 was calculated as (0 x percentage of tumor cells staining 0+) + (1 x percentage of tumor cells staining 1+) + (2 x percentage of tumor cells staining 2+) + (3 x percentage of tumor cells staining 3+), whereas the H-score of TS was calculated by the latter plus (4 x percentage of tumor cells staining 4+). A H-score ≤ median was assessed as ERCC1 negative; a H-score higher than the median was regarded as ERCC1 positive.

Twenty-eight biopsies were evaluable for analysis, as biopsies of twelve patients were not available. Twenty-seven resections were evaluable for analysis, as tissue of seven patients was not available, four patients had a complete response following nCRT, and for two patients no representative slides of the tumor were available.

1.5 Proteomics

A quality control was performed in the 92-plex proximity-extension assay by using an internal extension control and an inter-plate control. All values were normalized and presented as Normalized Protein Expression (NPX) values, in which higher NPX values correspond to higher protein expression. Individual samples were excluded if they failed quality control, and proteins were excluded if ≥40% of samples were below the lower limit of detection.

Machine learning analysis were performed using XGBoost, as this method provides transparency in determining the biomarkers with the highest relevance in predicting the outcome, in contrast to, for instance, Neural Networks.

The R2 genome analysis and visualization platform (http://r2.amc.nl) was used to identify differentially expressed proteins between patients with an event (deceased) and without an event (alive). A heatmap was created from the differentially expressed proteins.

1.6 Pharmacokinetic Analysis

Data were excluded if ≤3 measurements above the lower limit of detection were available per patient in one cycle. Measurements below the limit of detection were set to zero. If post-Tmax samples had 5-FU concentrations below the lower limit of detection, these values were inferred at half of the lower limit of detection. This technique was applied in order to reduce selection bias of only patients with the highest concentrations (above the lower limit of detection).[2] We compared the original pharmacokinetic data set, in which values below the lower limit of detection were set to zero, and the data set in which we incorporated the lower limit of detection divided by two technique, and found no significant changes between both datasets.

Appendix 2: Supplementary Figures

Supplementary Figure S1. *Treatment Exposure of S-1 and Oxaliplatin. Dose completion was defined as administration of all preplanned doses of S-1 and oxaliplatin without a dose reduction, dose interruption, or dose delay.*

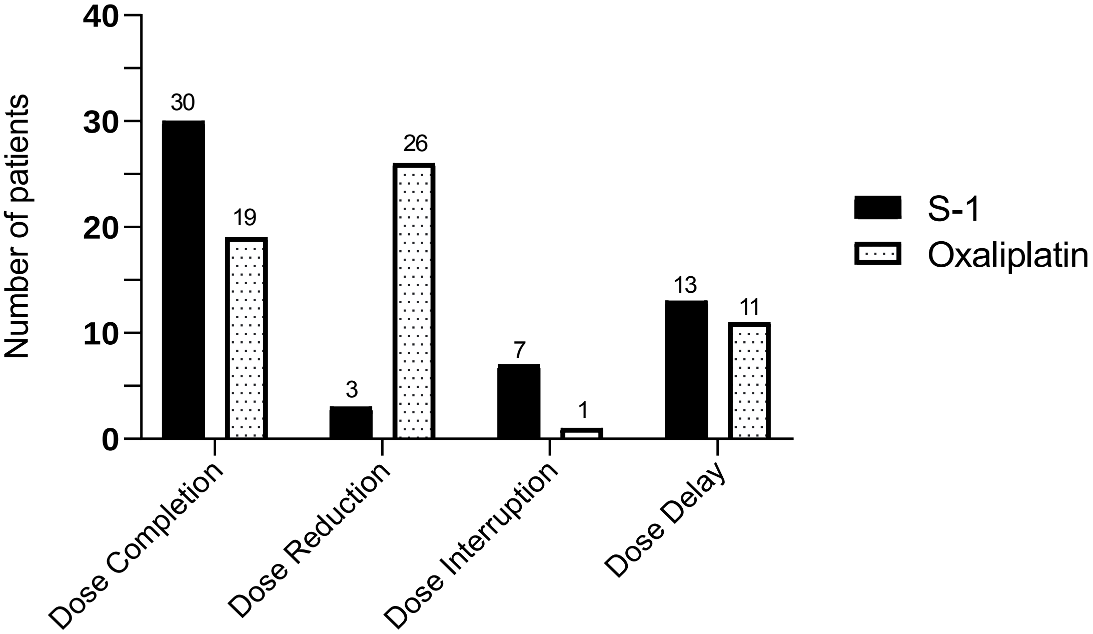

Supplementary Figure S2. Multivariate Subgroup Analysis in the SOX study.

Variables	N (SOX; NCR)	Hazard Ratio (95% CI)	Pinteraction
Differentiation Grade			0.01
1-2	103 (23; 80)	0.172 (0.051 - 0.576)	
3-4	75 (16; 59)	1.248 (0.611 - 2.549)	
Resected Positive Lymph nodes			0.05
<4	162 (35; 127)	0.104 (0.017 - 0.629)	
≥4	23 (5; 18)	0.722 (0.387 - 1.345)	
Mandard			0.87
1-2	69 (17; 52)	0.609 (0.219 - 1.690)	
3-4	111 (23; 88)	0.601 (0.292 - 1.237)	
pT stage			0.48
0-2	81 (17; 64)	0.372 (0.108 - 1.275)	
3	104 (23; 81)	0.695 (0.352 - 1.373)	

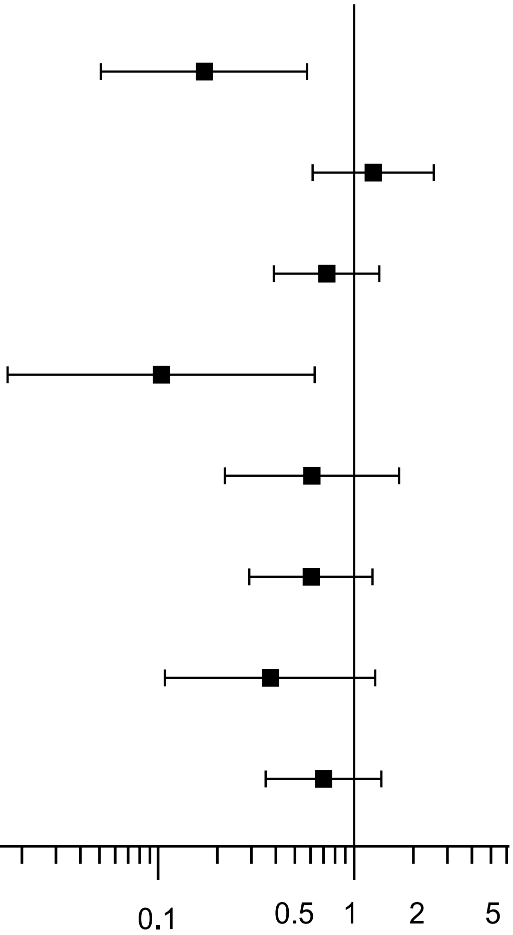

Supplementary Figure S3. ERCC1 immunohistochemistry expression at 20x magnification. (A) H-score of 140; (B) H-score of 180; (C) H-score of 250; (D) H-score of 300

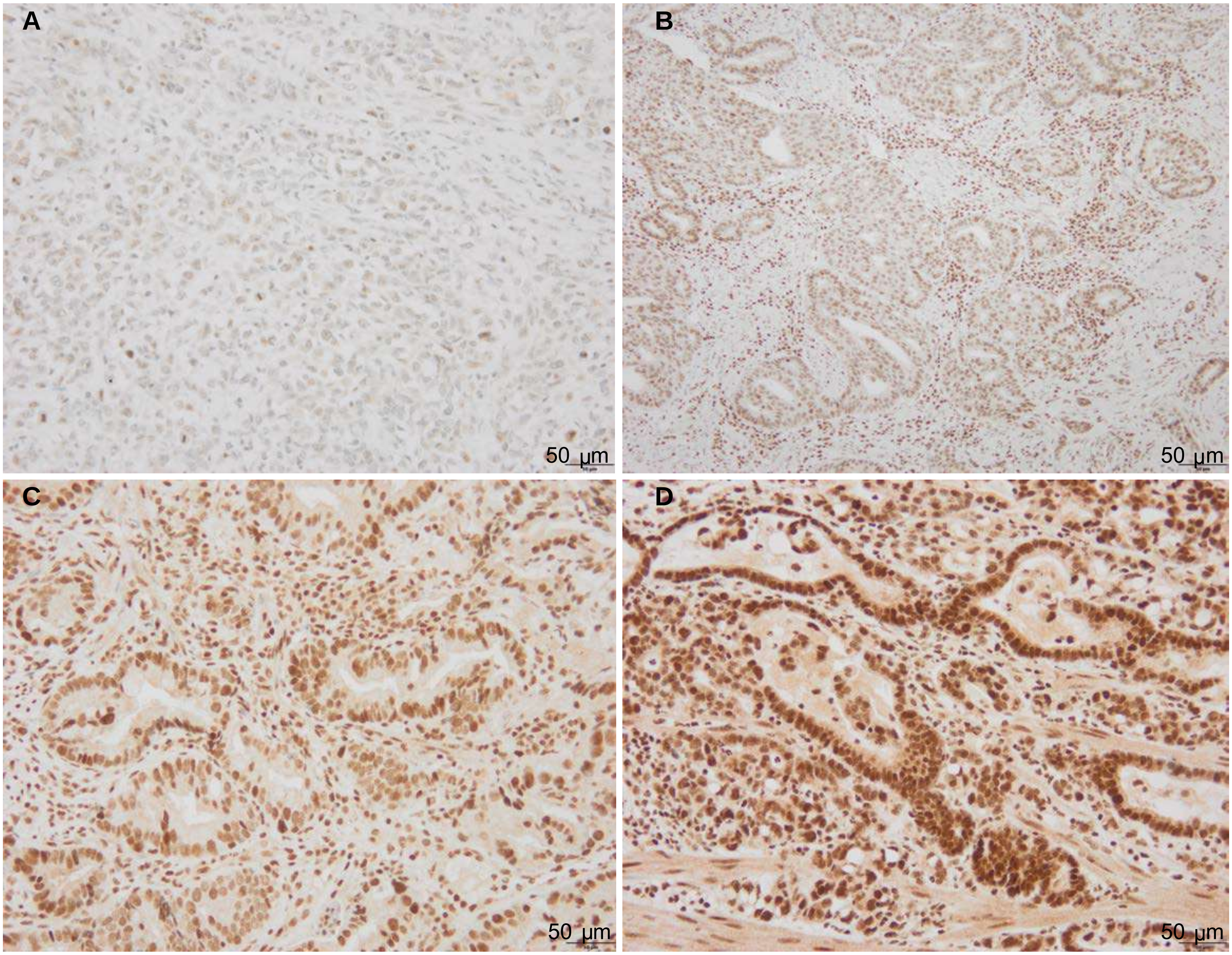

Supplementary Figure S4. TS immunohistochemistry expression at 20x magnification. (A) H-score of 30; (B) H-score of 100; (C) H-score of 200; (D) H-score of 300

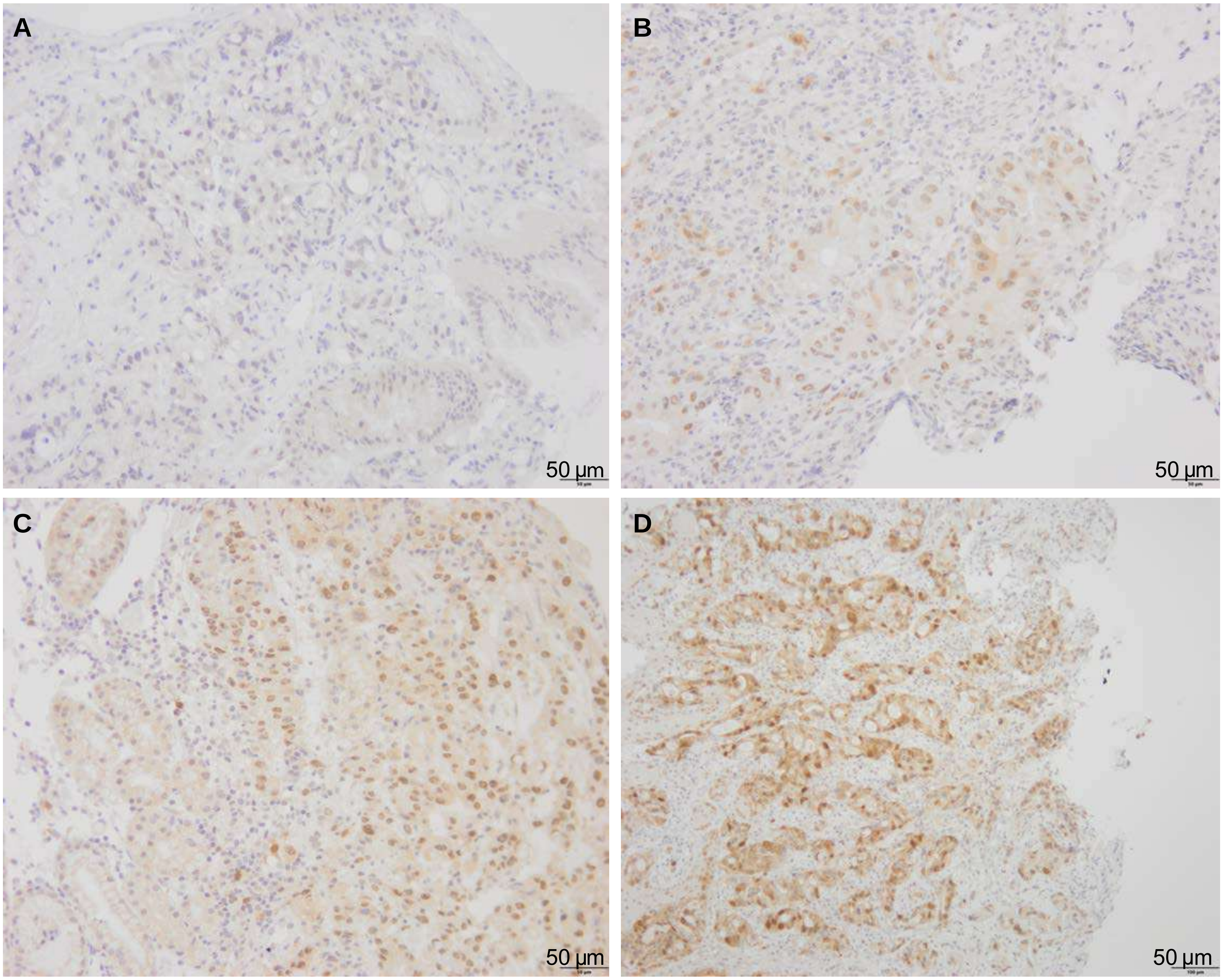

Supplementary Figure S5. Survival of patients with TS negative resection specimens vs. TS positive resection specimens.

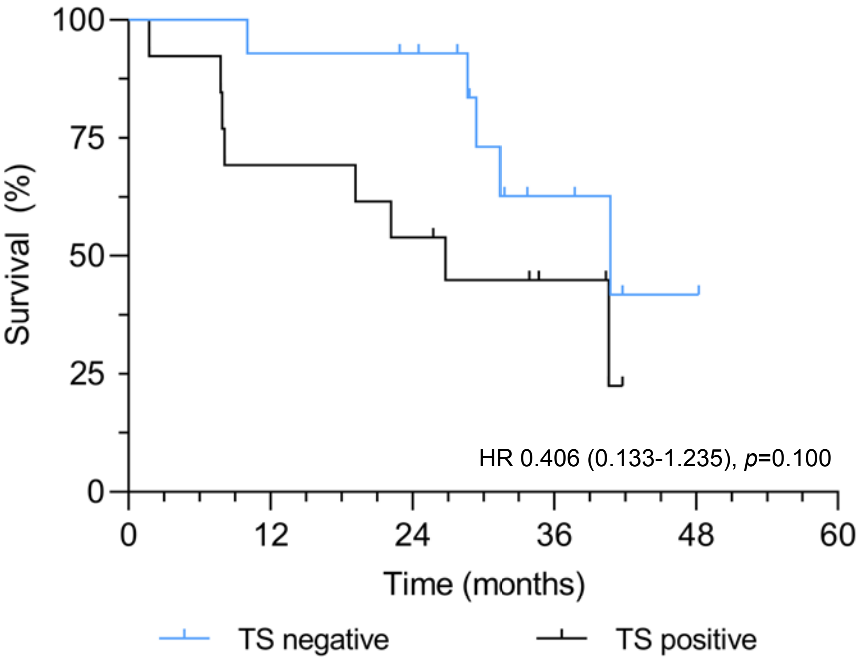

No. at risk

TS negative	14	14	13	5	2
TS positive	13	10	8	4	1

Supplementary Figure S6. Heatmap of differentially expressed proteins between dead patients vs. alive patients

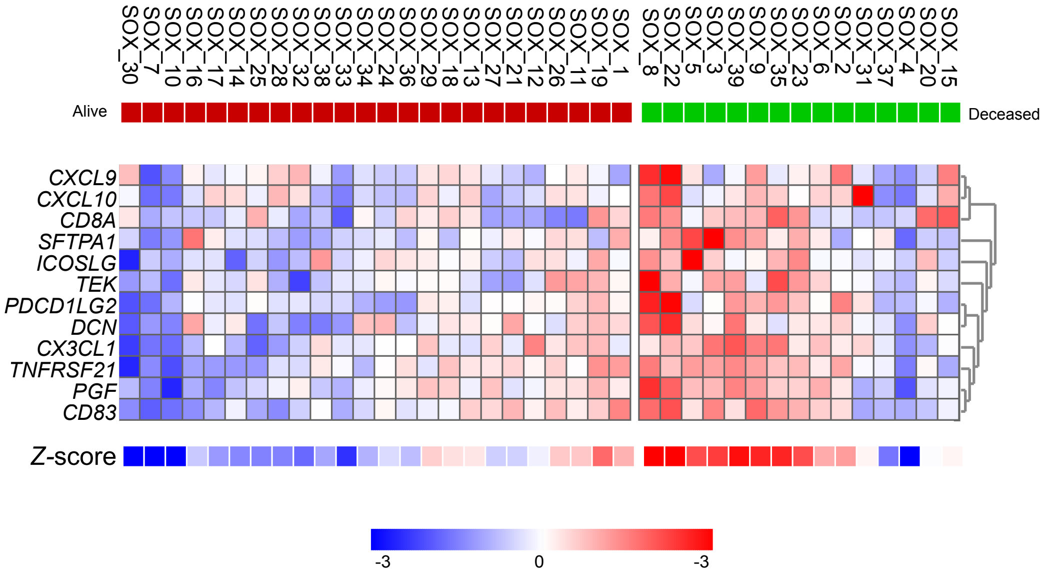

Appendix 3: Supplementary Tables

Supplementary Table S1. Propensity-score Matching Details of the SOX cohort and the Netherlands Cancer Registry (NCR) cohort. Variables were compared using a Chi-Square test or Fisher's exact test, as appropriate. *To safeguard anonymity, only the p-value has been given for the resection volume of the performing hospitals. SD denotes standard deviation.

Variable	SOX (n=40)	NCR (n=145)	*P*-value
Age			0.352
Median	60	60	
Interquartile Range	53-63	56-65	
Sex			0.214
Male	37 (93%)	132 (91%)	
Female	3 (8%)	13 (9%)	
Tumor Location			0.636
Distal Esophagus	29 (73%)	118 (81%)	
Junction	11 (28%)	27 (19%)	
Tumor Length			0.095
Mean	6.9	6.1	
SD	3.55	3.16	
Type of resection			0.323
Transhiatal	3 (8%)	11 (8%)	
Transthoracic with intrathoracic anastomosis	33 (83%)	113 (78%)	
Transthoracic with cervical anastomosis	4 (10%)	21 (14%)	
ypT stage			0.804
0	4 (10%)	0 (0%)	
1	7 (18%)	39 (27%)	
2	6 (15%)	25 (17%)	
3	23 (58%)	81 (56%)	
ypN stage			0.143
0	19 (48%)	65 (45%)	
1	14 (35%)	53 (37%)	
2	4 (10%)	20 (14%)	
3	3 (8%)	7 (5%)	
Tumor Grade			0.548
1	2 (5%)	7 (5%)	
2	21 (53%)	73 (50%)	
3	14 (35%)	59 (41%)	
4	2 (5%)	0 (0%)	
X	1 (3%)	0 (0%)	
Unknown	0 (0%)	6 (4%)	
Comorbidities			0.403
0	14 (35%)	54 (37%)	
1	15 (38%)	51 (35%)	
≥2	11 (28%)	40 (28%)	
Hospital Volume			0.812

Supplementary Table S2. Details of the SOX cohort and Matched cohort from archival material for ERCC1 immunohistochemistry. Variables were compared using non-parametric statistical testing. IQR denotes interquartile range.

Variable	SOX	Matched cohort	*P*-value
Age			0.671
Median (IQR)	62 (57-65)	63 (55-68)	
Sex			>0.999
Male	29	29	
Female	1	1	
ypT stage			0.878
1	7	6	
2	4	5	
3	19	19	
ypN stage			0.756
0	13	12	
1	12	12	
2	4	5	
3	1	1	
Mandard score			0.368
2	12	12	
3	13	9	
4	5	7	
5	0	2	

Supplementary Table S3. Description of proteins included in the proteomics machine learning model.

Protein	Full name	Function
NCS1	Neuronal Calcium Sensor 1	Regulator of G protein-coupled receptor phosphorylation in a calcium dependent manner. Regulated GRK1.
CXCL1	Growth-regulated alpha protein/C-X-C motif chemokine 1	Elicits chemotactic activity on neutrophils and among others is involved in inflammatory response.
GPA33	Cell surface A33 antigen	May play a role in cell-cell recognition and signaling
CXCL5	C-X-C motif chemokine 1	Elicits chemotactic activity on neutrophils. Activated upon interleukin-1 and TNF-alpha stimulation.
CSF-1	Macrophage colony-stimulating factor 1	Cytokine and a growth factor that functions as an inducer of proliferation and differentiation of hematopoietic stem cells to macrophages and monocytes.
MCP-4 (CCL13)	Monocyte chemotactic protein 4	Elicits chemotactic activity in monocytes, eosinophils, T lymphocytes, and basophils. Can be induced by pro-inflammatory cytokines IL-1 and TNF-alpha
CD27	CD27 antigen	Member of the tumor necrosis factor receptor superfamily, and is needed for generation and maintenance of T cell immunity. Plays a key role in regulating B-cell activation and immunoglobulin synthesis.
VEGFR2	Vascular endothelial growth factor receptor 2	Acts as a cell-surface receptor for VEGFA, VEGFC and VEGFD. Plays an essential role in the regulation of angiogenesis, vascular development, vascular permeability, and embryonic hematopoiesis. Promotes proliferation, survival, migration and differentiation of endothelial cells.
GZMB	Granzyme B	Expressed by cytotoxic T lymphocytes (CTL) and natural killer (NK) cells.
IL18	Interleukin-18	Pro-inflammatory cytokine that in combination with IL-12 induces cell-mediated immunity in the presence of microbial infection, which leads to the release of IFN-gamma
CCL23	C-C motif chemokine 23	Chemokine with highly chemotactic activity for resting T cells and monocytes
NELL1	Protein kinase C-binding protein NELL1	Plays a role in the control of cell growth and differentiation. Promotes osteoblast cell differentiation and terminal mineralization.
CXCL11	C-X-C motif chemokine 11	Elicits chemotactic activity on interleukin-activated T cells and it is also involved in inflammatory response. CXCL11 is not constitutively expressed but is up-regulated in a pro-inflammatory cytokine environment and is highly induced by IFN-beta and IFN-gamma.
IFN-y	Interferon gamma	Cytokine that plays critical roles in innate and adaptive immunity against viral and bacterial infections as well as tumor control.
CCL19	C-C motif chemokine 19	May play a role in normal lymphocyte recirculation and homing as well as in inflammatory and immunological responses

REFERENCES SUPPLEMENT

1. Mandard A‐M, Dalibard F, Mandard J‐C, Marnay J, Henry‐Amar M, Petiot J‐F, et al. Pathologic assessment of tumor regression after preoperative chemoradiotherapy of esophageal carcinoma. Clinicopathologic correlations. Cancer. 1994;73(11):2680–6.

2. Keizer RJ, Jansen RS, Rosing H, et al: Incorporation of concentration data below the limit of quantification in population pharmacokinetic analyses. Pharmacol Res Perspect 3:1–15, 2015
